# Biosafety and biosecurity as essential pillars of international health security and cross-cutting elements of biological nonproliferation

**DOI:** 10.1186/1471-2458-10-S1-S12

**Published:** 2010-12-03

**Authors:** Lela Bakanidze, Paata Imnadze, Dana Perkins

**Affiliations:** 1 National Center for Disease Control and Public Health of Georgia, Tbilisi, Georgia; 2 U.S. Department of Health and Human Services, Office of the Assistant Secretary for Preparedness and Response, Washington DC, USA

## Abstract

The critical aspects of biosafety, biosecurity, and biocontainment have been in the spotlight in recent years. There have also been increased international efforts to improve awareness of modern practices and concerns with regard to the safe pursuit of life sciences research, and to optimize current oversight frameworks, thereby resulting in decreased risk of terrorist/malevolent acquisition of deadly pathogens or accidental release of a biological agent, and increased safety of laboratory workers. Our purpose is to highlight how the World Health Organization’s (WHO) revised International Health Regulations (IHR[2005]), the Biological Weapons Convention (BWC), and the United Nations Security Council Resolution (UNSCR) 1540 overlap in their requirements with regard to biosafety and biosecurity in order to improve the understanding of practitioners and policymakers and maximize the use of national resources employed to comply with internationally-mandated requirements. The broad range of goals of these international instruments, which are linked by the common thread of biosafety and biosecurity, highlight their significance as essential pillars of international health security and cross-cutting elements of biological nonproliferation. The current efforts of the Republic of Georgia to enhance biosafety and biosecurity in accordance with these international instruments are summarized.

## Introduction

Natural outbreaks of disease could pose significant challenges to global security by undermining national economies, international trade and travel, public health and safety, and the trust of populace in its own government, potentially leading to ineffective governance or fragile state collapse. The global biological threat environment is compounded by the possibility of rogue states and/or terrorists deliberately using biological agents as weapons of war. Any such use of a biological agent (whether overtly or covertly) could have potentially devastating consequences on public health or the environment. Achieving effective, comprehensive biosecurity to prevent unauthorized possession, loss, theft, misuse, diversion, or intentional release of biological agents and toxins is a shared responsibility at the international level since infectious disease knows no borders.

Biosafety is complementary to biosecurity, and refers to the implementation of laboratory practices and procedures, specific construction features of laboratory facilities, safety equipment, and appropriate occupational health programs when working with potentially infectious microorganisms and other biological hazards. These measures are designed to reduce the exposure of laboratory personnel, the public, agriculture, and the environment to potentially infectious agents and other biological hazards. Laboratory-acquired infections (LAIs) have also started to receive more attention in recent years, in particular with regard to high (biosafety level 3, or BSL-3) and maximum (BSL-4) containment laboratories. LAIs may occur in research labs, clinical labs, or animal facilities, and sometimes it is difficult to determine whether the infection was acquired in the lab or from the community. There is also a strong public health concern related to the LAIs, as an infected laboratory worker may transmit the infectious disease to his colleagues, family, or community at large [1]. Poor personnel training increases the risk of a LAI or other biological accident in the laboratory, and may also contribute to improper pathogen accounting, storage and transportation, which in turn could contribute to the illicit acquisition of biological agents by terrorists or would-be bio-criminals.

**Figure 1:**
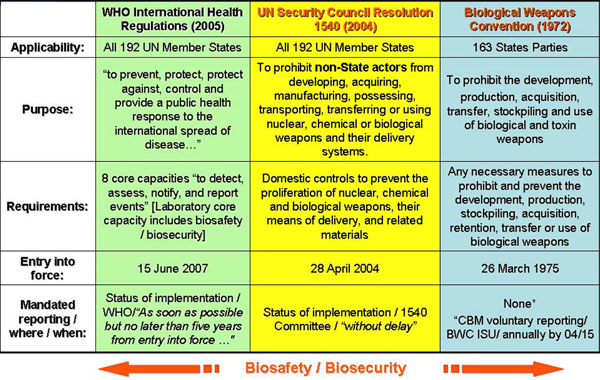
Biosafety and biosecurity are essential pillars of international health security and cross-cutting elements of biological nonproliferation.

Since there is no single technology or process that could be applied to prevent or deter the use of biological agents as weapons, the implementation of international instruments for nonproliferation (such as the Biological Weapons Convention and United Nations Security Council Resolution 1540) and public health (such as the International Health Relations) summarized in Figure 1, as well as the establishment of regional and international partnerships in countering biological threats (whether natural, accidental or deliberate in nature), are critical factors in achieving global health security. The pillars supporting the global health security are biosafety and biosecurity as they transcend unique national concerns and stand at the nexus of public health and security. This paper discusses each of these international instruments in detail, and then presents how Georgia is using these instruments to promote biosafety and biosecurity.

## Biosafety and biosecurity under the International Health Regulations (2005)

The International Health Regulations (IHR), the legally-binding international agreement designed to prevent the spread of disease, were revised and adopted in their new form by the 58^th^ World Health Assembly (WHA) on 23 May 2005. The purpose and scope of the IHR(2005) are “to prevent, protect against, control and provide a public health response to the international spread of disease in ways that are commensurate with and restricted to public health risks, and which avoid unnecessary interference with international traffic and trade.” [2]. The revised IHR apply to diseases (including those with new and unknown causes), irrespective of origin or source, that present significant harm to humans, and offer the international community new opportunities to strengthen the public health capacities and collaborate with other countries and with the World Health Organization (WHO).

Following the entry into force of the IHR(2005) in 2007, States Parties are required to meet the core capacity requirements as soon as possible, but no later than five years from the entry into force of the Regulations. As of 15 June 2007, States Parties had two years to assess their national structures and resources and develop national action plans, and as of 15 June 2009, States Parties have three years to meet the core capacity requirements. Core capacity 8, the laboratory core capacity, refers to those laboratory quality services relying on communication, specimen collection and transport, financial resources, biosafety and biosecurity best practices, trained personnel, suitable infrastructure, appropriate equipment and reagents, and the delivery of reliable results.

The WHO also developed a framework for States Parties to monitor the development of the 8 core capacities (through assessment and implementation), which includes a checklist of indicators that can be used for annual reporting on the IHR implementation to the WHA in accordance with Article 54.1 of the Regulations, and also for better targeting of WHO and Partner support to countries (see Table 1) [3]. These indicators are also meant to provide information about areas of focus for improvement and inform the strategic planning via a feedback process. Specifically, the framework provides: i) a set of 20 global indicators for monitoring the development of IHR core capacities for annual reporting to the WHA by all States Parties (mandatory for all); and ii) an additional 10 indicators for monitoring the comprehensive development, strengthening, and maintenance of States Parties’ IHR core capacities (optional).

**Table 1:** IHR (2005) checklist of indicators for annual reporting to WHA. Biosafety and biosecurity are included under indicator 13.

20 indicators for annual reporting to WHA
1. Laws, regulations, administrative requirements, policies or other government instruments in place are sufficient for implementation of obligations under the IHR.
2. A mechanism is established for the coordination of relevant sectors in the implementation of the IHR.
3. IHR National Focal Point (NFP) functions and operations are in place as defined by the IHR(2005).
4. Indicator-based routine surveillance includes an early warning function for the early detection of public health events.
5. Event-based surveillance is established.
6. Public health emergency response mechanisms are established.
7. Infection prevention and control (IPC) is established at national and hospital levels.
8. A multi-hazard National Public Health Emergency Preparedness and Response Plan has been developed.
9. Public health risks and resources are mapped.
10. Mechanisms for effective risk communication during a public health emergency are established.
11. Human resources are available to implement IHR core capacity requirements.
12. Laboratory services to test for priority health threats are available and accessible.
13. Laboratory biosafety and biosecurity practices are in place.
14. Effective surveillance is established at Points of Entry (PoE).
15. Effective response is established at PoE.
16. General obligations at PoE are fulfilled.
17. Mechanisms are established for detecting and responding to zoonoses and potential zoonoses.
18. Mechanisms are established for detecting and responding to foodborne disease and food contamination.
19. Mechanisms are established for detection, alert and response to chemical emergencies.
20. Mechanisms are established for detecting and responding to radiological and nuclear emergencies.

Building laboratory capacity to support a public health system cannot be done effectively without a strong focus on biosafety. The WHA had highlighted this issue in several resolutions, listed in Table 2.

**Table 2:** WHA resolutions on biosafety.

WHA resolutions
• World Health Assembly resolution 55.16 (2002):
“Global public health response to natural occurrence, accidental release or deliberate use of biological and chemical agents or radionuclear material that affect health”
• World Health Assembly resolution 58.3 (2005):
“Prevention and control of the international spread of disease and public health risks”
• World Health Assembly resolution 58.29 (2005):
“Enhancement of laboratory biosafety”

A national health security strategy intended to protect the population against public health emergencies must consider a diverse spectrum of threats, including endemic diseases, natural outbreaks or pandemics, accidents involving biological agent release, bioterrorism attacks, and biological warfare, all of them having a wide range of potential consequences. Whether preparing for a natural or a deliberate event, the common denominator is the need for a robust and timely response, and adaptive public health system that will provide early warning and an efficient medical response.

Implementation of the consistent policies, operating procedures and the operational and technical capacity required by the IHR(2005) will help ensure early warning and efficient international management of a biological incident, whether naturally occurring or deliberate in nature, thereby promoting our national health security. Laboratory-based surveillance and outbreak detection are essential to the prevention and mitigation of biological threats, and quality laboratory services are dependent on the implementation of biosafety and biosecurity best practices supported by an appropriate legal framework.

## Biosafety and biosecurity under the Biological Weapons Convention

The Biological Weapons Convention (BWC), formally known as the *Convention on the Prohibition of the Development, Production and Stockpiling of Bacteriological (Biological) and Toxin Weapons and on Their Destruction* (aka the Biological and Toxin Weapons Convention), was the first multilateral disarmament treaty that banned the production and use of an entire category of weapons. It was opened for signature on 10 April 1972, and entered into force on 26 March 1975 [4].

The BWC States Parties hold Review Conferences every five years (1980, 1986, 1991, 1996, 2001, and 2006, with the next one to be held in 2011). Between these Review Conferences, States Parties have pursued various activities and initiatives to strengthen the effectiveness and improve the implementation of the Convention. For example, the 2006 BWC Sixth Review Conference created the 2007-2010 intersessional process, which consists of four sets of annual meetings prior to the Seventh Review Conference (each set includes a one-week Meeting of Experts, followed by a one-week Meeting of States Parties); established the Implementation Support Unit (ISU); established an action plan for universalization and improving national implementation; improved the Confidence Building Measures (CBM) information exchange process; worked on enhancing provisions of assistance; and built a network of national points of contact.

CBMs were first agreed upon at the Second Review Conference in 1986 “in order to prevent or reduce the occurrence of ambiguities, doubts and suspicions and in order to improve international co-operation in the field of peaceful biological activities.” [5]. The CBMs were modified and considerably expanded in 1991. They have not been modified since, though it is expected that the Seventh Review Conference in 2011 will undertake a significant review of current CBM forms and content.

The CBMs involve voluntary exchanges of information on a range of BWC-related activities, including research centers and laboratories, national biological defense research and development programs, vaccine production facilities, and unusual outbreaks of infectious diseases. Since the CBMs are not legally-binding (i.e., not required by any article of the Convention), but established only as voluntary (politically-binding) measures, participation in the CBMs is not universal or consistent from year to year.

In order to ensure that the tenets of the BWC are adhered to, States Parties are encouraged to implement national legislation to enforce the provisions of the BWC to prohibit and prevent the development, production, stockpiling, acquisition, retention, transfer or use of biological weapons by anyone under their jurisdiction, as well as parallel measures to prohibit and prevent encouraging, inciting or assisting others in any of these acts. However, the precise details of what measures are necessary to accomplish these goals and implement the provisions of the Convention are at the discretion of individual States Parties.

Based on the understandings and agreements reached historically at the Review Conferences, national imple-mentation of BWC includes legislative, administrative, and other measures to enhance domestic compliance with the BWC; national export control systems; education, awareness raising and outreach measures; disease surveillance, detection, and containment; as well as biosafety and biosecurity provisions.

In this context, the common understandings reached at the 2008 BWC Meeting of States Parties are highly relevant: “recognizing that biosafety and biosecurity measures contribute to preventing the development, acquisition or use of BTW [biological and toxin weapons] and are appropriate means of implementing the BWC, States Parties agreed on the value of…international cooperation on biosafety and biosecurity at the bilateral, regional and international levels,” and also that “pursuing biosafety and biosecurity measures could also contribute to the fulfillment [by State Parties] of other respective international obligations and agreements, such as the revised IHR of the WHO, and relevant codes of OIE [the International Organization for Animal Health],…[and] UNSCR [United Nations Security Council Resolution] 1540 (2004) that places obligations on all states and is consistent with the provisions of the Convention.” [6].

While the understandings and agreements reached during the intersessional process are not legally-binding, they are nevertheless politically-binding for all States Parties. States Parties have the opportunity to report under the CBM E (Declaration of legislature, regulations, and other measures) the relevant laws, regulations, or other measures related to the national biosafety and biosecurity framework. Additionally, the CBM D (Active promotion of contacts) also offers an opportunity for States Parties to promote relevant educational and training activities in these areas.

## United Nations Security Council Resolution 1540 (UNSCR 1540)

On 28 April 2004, the UN Security Council unanimously adopted UN Security Council Resolution 1540 (UNSCR 1540) to address the risk that terrorists and illicit networks will acquire Weapons of Mass Destruction (WMD). UNSCR 1540 established for the first time legally-binding obligations on all UN Member States to develop and to enforce effective measures against the proliferation of nuclear, chemical, and biological WMD, their means of delivery, and related materials. While national implementation efforts under the BWC, Chemical Weapons Convention (CWC), and the Treaty on the Non-Proliferation of Nuclear Weapons (NPT) are intended to accomplish a similar goal, 1540’s sole intention is to create broad-range binding obligations regard ing all three weapon types and avoid the negotiation processes and voluntary commitments under these treaties. Moreover, it is applicable to all UN Member States, regardless of their membership in multilateral agreements.

The resolution calls for the establishment of a national legal framework that should include the following elements:

• A system to account for and secure items in production, use, storage or transport;

• Effective physical protection measures;

• Effective border controls and law enforcement measures; and

• Effective national export and trans-shipment controls. UNSCR 1540 also emphasizes that the international legal framework facilitate a strategy of “prevention” based upon each individual State accepting “responsibility” for implementing measures against the proliferation of materials and weapons. This is why UNSCR 1540 also requires all States to report on their national implementation measures to the 1540 Committee established pursuant to the resolution. UNSCR 1673 (27 April 2006), renewed the 1540 Committee for two additional years. In its resolution 1810 (2008), the Council decided to extend further the mandate of the Committee for a period of three years until 25 April 2011, to continue to promote the full implementation by all States of resolution 1540 (2004) through its program of work, which includes the compilation of information on the status of States’ implementation of all aspects of resolution 1540 (2004), outreach, dialogue, assistance and cooperation. Per UNSCR 1810 (2008), the Committee would submit to the Council a report no later than 24 April 2011 on compliance with resolution 1540 (2004) through the achievement of the implementation of its requirements.

States were asked to submit a first report, not later than six months after the adoption of the resolution 1540, (i.e. 28 October 2004), on steps they had taken or intended to take to implement this resolution. As of 1 July 2008, the total number of States that had submitted at least one report since 2004 stood at 155 (out of the 192 UN Member States). Of those States that had submitted first reports, 102 submitted additional information. Thirty-seven States have not submitted a first report to the Committee. The 1540 Committee also acts as a clearing house for information on the issue of assistance through formal and informal contact and dialogue with all States, especially those expressing interest in offering and receiving assistance. The 1540 Committee developed matrices to be used as tools for dialogue with States on their implementation of the resolution, as well as for facilitating technical assistance. A matrix for each UN Member State has been prepared. The matrices are regularly updated and approved by the Committee.

For example, the matrix for biological weapons and related materials identifies the following areas where domestic controls should be implemented and enforced:

• Measures to account for/secure production

• Measures to account for/secure use

• Measures to account for/secure storage

• Measures to account for/secure transport

• Regulations for physical protection of facilities/materials/transports

• Licensing/registration of facilities/persons handling biological materials

• Reliability check of personnel

• Measures to account for/secure/physically protect means of delivery

• Regulations for genetic engineering work

• Other legislation/regulations related to safety and security of biological materials

In response to UNSCR 1810 (2008), the 1540 Committee conducted in the Fall of 2009 a Comprehensive Review as a forum for all States and relevant intergovernmental bodies to share experiences and express their views on various aspects of UNSCR 1540 implementation and also (i) to assess the evolution of risks and threats; (ii) to address specific critical issues that have not yet been resolved; and (iii) to identify possible new approaches for the implementation of the resolution. The broad participation during the Comprehensive Review included formal statements and interventions on specific issues made by 41 States and 21 intergovernmental organizations and other entities. Based on the findings of the Comprehensive Review, the 1540 Committee prepared an outcome document with recommendations to the Council regarding the implementation of Resolution 1540. This document acknowledged the significant number of measures that States have taken to implement 1540 obligations, but identified some areas in which States have adopted fewer measures, such as biological weapons, means of delivery, national control lists, and access to related materials and financing of prohibited or illicit proliferation activities [7].

Areas covered under the UNSCR 1540-required regulatory framework overlap with Georgia’s efforts on strengthening the current biosafety, biosecurity, and biocontainment oversight frameworks aimed at decreasing the risk of terrorist/malevolent acquisition of deadly pathogens or accidental release of a biological agent. However, the bioterrorism prevention in the context of UNSCR 1540 requires continued international support toward the shared goals of achieving international health security and prohibiting biological nonproliferation.

## Georgia’s efforts on strengthening national biosafety and biosecurity

In order to be comprehensive and ensure an effective implementation, a national legislative system on biosafety and biosecurity has to be considered in the context of other pertinent legislation and extant measures, and should have “buy-in” from all relevant stakeholders. In Georgia, such stakeholders include the Ministry of Labor, Health and Social Affairs (MOHLSA); the Ministry of State Security; the Ministry of Interior; and the Ministry of Infrastructure.

Ensuring biosafety and biosecurity in Georgia is one of the main responsibilities of the National Center for Disease Control and Public Health (NCDC), which comprises a network of 11 regional and 66 district (rayon) Centers for Public Health and also houses the Georgian national collection of especially dangerous pathogens. NCDC was built on the foundation of the Georgian Station for Plague Control in 1996 and its statute was approved by the President of Georgia by Presidential Decree 55 on 21 February 2003. NCDC now employs 440 personnel (60% are specialists with graduate-level education).

The designation of NCDC as the National Focal Point for the IHR provided a strong renewal of commitment to advance the legislative framework for biosafety and biosecurity in Georgia in the context of the national efforts to meet the core capacity requirements of the IHR. Moreover, experts from Georgia are very active in collaborating with the WHO and other organizations and partners in technical consultations related to the IHR. For instance, Georgian experts participated in the technical consultation on checklist and indicators for monitoring progress in the implementation of IHR core capacities in Member States organized by WHO in Lyon, France, 4-6 August 2009.

Georgia joined the Biological Weapons Convention in 1995 and has extensive measures in place to ensure that all activities on its territory are treaty-compliant and that prohibited activities are deterred and detected and perpetrators are punished. The basic tenets and understandings reached in the BWC intersessional process are implemented by Georgia through:

• Legislation and regulations;

• Biosafety and biosecurity;

• Oversight of life sciences research;

• Education and awareness of dual use issues and biological risk;

• Disease surveillance, containment, and response.

In addition, Georgia participates in the CBM process (submitting eight annual reports since it ratified the treaty) and is actively involved in the BWC intersessional process (conducting joint presentations with the U.S. and UK at the Meeting of Experts in 2009 and a joint presentation with the U.S. on Southern Caucasus Partner ships in Countering Biological Threats in 2010). On the sides of the 2010 BWC Meeting of Experts, Georgia also presented at the First European Union Joint Action Workshop, on “Practicalities for BWC Implementation and Confidence Building Measures Reporting,” since technical assistance and exchanges of experience gained from preparing the annual CBM reports can increase compliance with voluntary reporting and strengthen the BWC through increased transparency and openness.

The strategic vision for an effective and comprehensive framework for biological risk management in Georgia (comprising biosafety and biosecurity) involves a set of regulations on biosecurity (based on the U.S. Select Agents Rule and similarly covering facilities and person-nel registration, security risk assessments, emergency response, record keeping, inspections, duties of Responsible Official, training, notifications for theft, loss or release, etc); biosafety norms (consistent with the “Bio-safety in Microbiological and Biomedical Laboratories” guidance published by the U.S. Centers for Disease Control and Prevention [CDC] and the WHO “Laboratory Biosafety Manual”); regulations for import, export, containment, transfer, and handling of biological agents and toxins; and guidelines for safe transportation of infectious substances and diagnostic materials.

To that end, and in accordance with the NCDC statute which specifies “participation in preparing normative and methodological documentation under its competen-cies,” experts from the NCDC Department of Biosafety and Threat Reduction and other institutions of MOHLSA have prepared a draft model law with the components mentioned above, in consultation with personnel from the U.S. Department of Health and Human Services (HHS), U.S. Department of Defense (DoD), and U.S. Department of State. However, this effort could only partly be completed since other pertinent legislative efforts should be pursued in parallel (for instance those regarding the criminal code and also the administrative code of Georgia, which will contribute to deterrence by increasing the penalties for misuse, theft, and diversion of biological agents). A close collaboration among the public health, law enforcement, the judicial branch and other stakeholders is necessary to ensure that the biological risk management framework is viewed holis-tically in the context of the national legislative system.

The recently revised legislation on public health (adopted on 27 June 2007) currently specifies in its Chapter V, “Providing Biosecurity/Biosafety,” the relevant measures, authorities and responsibilities in these areas, as follows:

• C1.16 – Providing Biosecurity/Biosafety;

• C1.17 – Limitation of Posession, Use, Transfer, Transportation and Destruction of Causative Agents of Especially Dangerous Infections;

• Cl.18 – Destruction of Causative Agents of Especially Dangerous Infections;

• Cl.19 – Import and Export of Causative Agents of Especially Dangerous Infections;

• Cl.20 – Institutions Responsibilities on Biosafety/Biosecurity;

• Cl.21 – Establishing a Unique Laboratory System for Detection, Surveillance and Response to Causative Agents of Especially Dangerous Infections.

In addition to drafting and implementing pertinent legislation, Georgia is collaborating with the United States on enhancing its biosafety and biosecurity by training its workforce and improving its biological infrastructure. The Defense Threat Reduction Agency (DTRA) is leading in Georgia the Cooperative Biological Engagement Program (CBEP) aimed at reducing the biological risk by securing/consolidating pathogens, training scientists in biosafety and biosecurity techniques, and regulatory reform; establishing a sustainable detection, response, and communication network to monitor biological outbreaks; and undertaking cooperative biological research projects to understand disease baseline, increase transparency, encourage higher ethics standards, and strengthen the integration of scientists into the international community.

Georgia is also closely collaborating with the U.S. Department of Health and Human Services. The CDC is working to help strengthen the public health systems of Georgia, Armenia, and Azerbaijan by improving each country’s disease detection response and control through improvements in laboratory systems, epidemiology workforce, and public health management skills. For instance, the South Caucasus Regional Field Epidemiology and Laboratory Training Program (FELTP) is based at NCDC in Tbilisi, Georgia, but also involves the neighboring countries of Armenia and Azerbaijan. The two-year in-service training program in applied epidemiology and public health laboratory practice trains residents in field epidemiology and public health laboratory for leadership positions in various levels of their respective ministries of health or agriculture. The FELTPs have a strong focus on biosafety and biosecurity.

Georgia supports the USNCR 1540 and submitted its report on national measures taken in implementation of its goals on 28 October 2004 with additional information provided to the 1540 Committee on 28 January 2006. The report outlined the legislative framework in Georgia; measures taken with regard to nonproliferation of chemical and biological weapons and disposal of radioactive sources; the introduction of Georgian system of export control of dual use materials, equipment and technologies; and the series of bilateral agreements with the United States on preventing the proliferation of WMD materials and technologies, counterterrorism, border security and export control. Georgia is also working on updating its legislation in order to cover all aspects of its obligations under the Resolution.

In addition to enhancing biosecurity and biosafety in Georgia through the IHR (2005), BWC and 1540 mechanisms, Georgia also supports the European Security Strategy (“A secure Europe in a better world”) and the European Union Strategy against the Proliferation of WMD (“Effective multilateralism, prevention and international cooperation”), adopted by the European Council on 12 December 2003, which identify proliferation as one of the five key challenges to international security, together with terrorism, regional conflicts, State failure, and organized crime.

Similarly, Georgia supports the North Atlantic Treaty Organization (NATO)’s “Comprehensive, Strategic-Level Policy for Preventing the Proliferation of WMDs and Defending against CBRN Threats” of 2009, which focuses on prevention and strengthening international non-proliferation mechanisms (i.e. BWC, UNSCR 1540, the Proliferation Security Initiative, etc.); and increased information exchange, engagement, cooperation, and joint training with Partner nations, international and regional organizations, and civilian entities.

### International workshops and training in Georgia

Under the auspices of NATO’s Science for Peace Program, Georgia organized in June 2008 a workshop on “Emerging and endemic pathogens: advances in surveillance detection, and identification,” which was attended by more than 50 experts from 10 countries (Georgia, U.S., UK, Russia, Ukraine, Kazakhstan, Macedonia, France, Germany and Azerbaijan).

Georgia also hosted and co-organized The Southern Caucasus Workshop on Public Health, Security, and Law Enforcement Partnership in Bio-Incident Pre-Planning and Response and the associated Southern Caucasus BioShield 2010 Tabletop Exercise (TTX) which were held in Tbilisi, Georgia, 11-12 May 2010. These events were a joint effort of DTRA, HHS’s Office of the Assistant Secretary for Preparedness and Response (ASPR), and Georgia’s NCDC [9].

Over 80 participants were in attendance at the May 2010 meeting, from inter-governmental organizations (WHO, International Criminal Police Organization [INTERPOL], NATO), U.S. Government (DoD, HHS, Department of Energy, Department of State, and Federal Bureau of Investigation [FBI]), and from public health, security, or law enforcement organizations from Georgia, Azerbaijan, Armenia, Kazakhstan, Moldova, and Romania. Non-governmental organizations such as VERTIC (Verification Research, Training and Information Centre), Bechtel, and Global Green USA also participated in these events.

The workshop and tabletop exercise aimed to:

• Foster improved understanding of the respective procedures and requirements of public health, security, and law enforcement communities in response to a biological incident, and enhance their joint effectiveness in pre-planning and response at the national and regional/international level;

• Enhance understanding of intergovernmental organizations’ role and their interaction in the process of sharing information and coordinating the international response;

• Emphasize the concept that information exchange in the early stages of a biological incident is critical to effectively containing the outbreak/mitigating the consequences of a biological incident and to apprehending the potential perpetrators;

• Review existing legal and regulatory infrastructure of national measures consistent with the obligations under the BWC, UNSCR 1540, and IHR(2005) to deter, prevent, or respond to biological incidents or threats.

These events successfully linked the international *response* to a bioterrorism incident stemming from the convergence of criminal and terrorist networks, with *prevention* via the nonproliferation mechanisms described in this paper:

• The BWC – by emphasizing the effective prohibition of the development, production, acquisition, transfer, retention, stockpiling and use of biological and toxin weapons and highlighting the treaty as a key element in the international community’s efforts to address the proliferation of WMD;

• UNSCR 1540 – by emphasizing the requirement that all UN Member States refrain from providing support to non-state actors that attempt to develop, acquire, manufacture, possess, transport or use nuclear, chemical or biological weapons and their means of delivery, and the obligation of Member States to establish and to enforce domestic controls to secure WMD-related materials and prevent their proliferation; and

• NATO’s *Comprehensive, Strategic-Level Policy for Preventing the Proliferation of WMDs and Defending against CBRN Threats* – by emphasizing its focus on prevention and strengthening international nonproli-feration mechanisms and increased information exchange, engagement, cooperation, and joint training with Partner nations, international and regional organizations, and civilian entities.

## Conclusion

The various international instruments described above are all part of the so-called “web of prevention” designed to address the multitude of security and health challenges of today’s world. Georgia is working toward building a culture of security and responsibility at the national and international level by involving civic, scientific, and government capacities in its outreach events to facilitate a common understanding of the WMD threat and encourage participation in and compliance with international arms control, disarmament and non-proliferation efforts; enhance global efforts to protect and defend against biological threats; and improve disease containment and response in case of outbreaks whether due to natural, accidental, or deliberate causes.

## Abbreviations

ASPR: Assistant Secretary for Preparedness and Response; BSL: Biosafety level; BTW: Biological and toxin weapons; BWC: Biological Weapons Convention; CBEP: Cooperative Biological Engagement Program; CBM: Confidence Building Measure; CDC: Centers for Disease Control and Prevention; CWC: Chemical Weapons Convention; DoD: Department of Defense; DTRA: Defense Threat Reduction Agency; FELTP: Field Epidemiology and Laboratory Training Program; FBI: Federal Bureau of Investigation; HHS: Department of Health and Human Services; IHR: International Health Regulations; INTERPOL: International Criminal Police Organization; IPC: Infection prevention and control; ISU: Implementation Support Unit; LAIs: Laboratory-acquired infections; MOLHSA: Ministry of Labor, Health, and Social Affairs; NATO: North Atlantic Treaty Organization; NCDC: National Center for Disease Control and Public Health; NFP: National Focal Point; NPT: Treaty on the Non-Proliferation of Nuclear Weapons; OIE: International Organization for Animal Health; PoE, Points of Entry; TTX: Tabletop Exercise; UNSCR: United Nations Security Council Resolution; WHA: World Health Assembly; WHO: World Health Organization; WMD: Weapons of Mass Destruction.

## Competing interests

The authors declare that they have no competing interests.

## Authors’ contributions

All authors contributed equally to this work.
